# Zeno dynamics in quantum open systems

**DOI:** 10.1038/srep11509

**Published:** 2015-06-23

**Authors:** Yu-Ran Zhang, Heng Fan

**Affiliations:** 1 Beijing National Laboratory for Condensed Matter Physics, Institute of Physics, Chinese Academy of Sciences, Beijing 100190, China; 2 Collaborative Innovation Center of Quantum Matter, 100190 Beijing, China

## Abstract

Quantum Zeno effect shows that frequent observations can slow down or even stop the unitary time evolution of an unstable quantum system. This effect can also be regarded as a physical consequence of the statistical indistinguishability of neighboring quantum states. The accessibility of quantum Zeno dynamics under unitary time evolution can be quantitatively estimated by quantum Zeno time in terms of Fisher information. In this work, we investigate the accessibility of quantum Zeno dynamics in quantum open systems by calculating noisy Fisher information when a trace preserving and completely positive map is assumed. We firstly study the consequences of non-Markovian noise on quantum Zeno effect and give the exact forms of the dissipative Fisher information and the quantum Zeno time. Then, for the operator-sum representation, an achievable upper bound of the quantum Zeno time is given with the help of the results in noisy quantum metrology. It is of significance that the noise reducing the accuracy in the entanglement-enhanced parameter estimation can conversely be favorable for the accessibility of quantum Zeno dynamics of entangled states.

Quantum Zeno effect (QZE), coined as the Zeno’s paradox in quantum theory, states that an unstable quantum system, if observed continuously, will never decay^1^. Hence we can slow down or even “freeze” the evolution of the system by frequent measurements in its known initial state. QZE is ascribed to two standard principles of quantum theory: continuous unitary time evolution in the absence of measurement and von Neumann projection postulate^2^. The state of the system need not remain frozen to its initial state, but it could evolve in a multidimensional subspace, called “Zeno subspace”, with measurement projecting on this subspace^3^. QZE is anticipated to have significant applications in protection of quantum states and creation of subspaces from decoherence provided by a variety of sources, which are urgent for robust quantum information processing^4^,^5^,^6^,^7^,^8^. There are also experimental studies attempting at the confirmation of QZE^9^ as well as its applications^10^,^11^. Experiments on QZE have been performed mainly for oscillating systems^12^,^13^, whilst there are several attempts to observe QZE in truly decaying states^14^,^15^.

QZE has become a focus of attention not only because it can be applied in robust quantum information processing, but also because of its foundational implications about the nature of quantum measurement^2^ as well as indistinguishability of state^16^ and entanglement^17^. Recently, it has been shown that Zeno dynamics can be comprehended as a physical consequence of the statistical indistinguishability of neighboring quantum states in Hilbert space^18^. For example we consider a system Hamiltonian driving a pure state 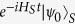, and *m* trials of projective measurements *M* = |*ψ*_0_〉_*S*_ 〈*ψ*_0_| are performed with equal time intervals *τ* = *t*/*m* during the dynamics. The survival probability to find the system at its initial state can be written as 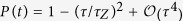 where *τ*_*Z*_ is the quantum Zeno time (ZT) in terms of Fisher information (FI) and equals to the largest interval such that two states remain indistinguishable^18^. Thus, the accessibility of quantum Zeno dynamics can be quantitatively estimated by ZT that is obtained by calculating FI. However, in real experiments there will always be some degree of noise and limitation. Zeno dynamics of nonunitary physical process in quantum open system deserves further investigation with fruitful results on the quantum Fisher information (QFI) in noisy systems^19^,^20^.

In quantum open systems, the dynamics of the system becomes “noisy” and nonunitary due to interaction with an environment. Generally, it can be described by a trace preserving and completely positive (CP) map, named as a quantum channel. Specifically, after the time unitary transformation *U*_*SE*_(*t*) acting on the state of system and environment *ρ*_*SE*_(0), we can obtain the reduced state of system alone after a partial trace over the environment 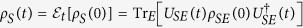
21. When we assume that the system-environment state is initially decoupled *ρ*_*S*_(0)⊗*ρ*_*E*_(0), the behavior of a quantum open system can be expressed by the operator-sum representation 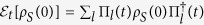 in terms of Karus operators. Moreover, in many cases it turns out to be useful to formulate the dynamics of an open system by means of a quantum Markovian master equation with Lindblad structure under the Born-Markovian approximation^22^. However, in many realistic physical systems the assumption of a Markovian dynamics relying on a number of mostly rather drastic simplifications is not sufficient for modern applicaitons and non-Markovian dynamics of an open system attracts nowadays increasing attention. Applying the time-convolutionless (TCL) projection operator technique^22^, we are able to obtain an exact master equation for the reduced system dynamics in which the non-Markovianity are considered.

In this work, we investigate the realizability of quantum Zeno dynamics in open system via judging the indistinguishability of state with noisy FI. We firstly investigate the consequences of non-Markovian noise on ZT via calculating noisy FI. Two exactly solvable models are considered. Then, we study the quantum Zeno dynamics in an open system expressed by operator-sum representation^21^. In this case, we can utilize the general manifestation of quantum Zeno dynamics of unitary process proposed in Ref. 18. An achievable upper bound of the ZT is deduced via calculations of QFI using the variational methods in noisy quantum metrology^20^. Furthermore, it has been shown in Ref. 18 that the entangled state may have a shorter ZT in unitary process than that of the separable state. We find that entangled state can have a ZT with a similar scale as that of the separable state by interacting with the a suitable model of open system. That is, the noise lowering the accuracy in the entanglement-enhanced parameter estimation can, on the contrary, be beneficial to the accessibility of quantum Zeno dynamics of entangled states.

## Results

### Dissipative Zeno dynamics via exact master equation

We consider an initial pure state *ρ*_*S*_(0) = |*ψ*_0_〉_*S*_〈*ψ*_0_| of a system *S* evolving under the impact of noise. For simplicity, we assume that the Hamiltionian for the system *H*_*S*_ is time independent and the dynamical equation describing the state is written in the interaction picture as 
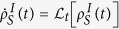
 and 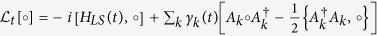. As usual we set *ħ* = 1. 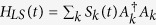 is the Lamb shift Hamiltonian, and {*A*_*k*_} is the set of Lindblad generators of the dynamical map. *S*_*k*_(*t*) is a time-dependent coefficient of the Lamb shift and *γ*_*k*_(*t*) denotes a time-dependent decay rate. In Markovian evolutions, we have *γ*_*k*_(*τ*) ≥ 0 ∀ *k* for *τ* ∈ [0, *t*], while if any *γ*_*k*_(*τ*) can be negative for some intervals, the dynamics of evolution will be non-Markovian^22^. Equivalently, the evolution in Schrödinger picture can be expressed as 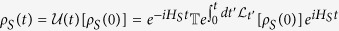, where 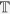 denotes time ordering.

We define the projective measurement applied in the quantum Zeno dynamics as 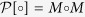 with *M* = |*ψ*_0_〉_*S*_〈*ψ*_0_|. A sequence of *m* observations can repeatedly bring the system to the initial state with survival probability 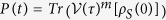, in which we define that 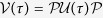 and the interval is *τ* = *t*/*m*. For the case of small time intervals *τ* ≪ *t* with a large enough number of trials *m *→ ∞, the survival probability can be expanded in terms of intervals *τ* as


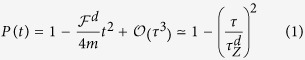


where


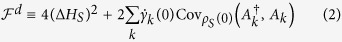


is called the dissipative Fisher information (d-FI)^23^ because 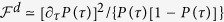. Here, Cov_*ρ*_(*X*, *Y*) ≡ Tr(*XYρ*) − Tr(*Xρ*)*Tr*(*Yρ*) is the covariance of observables *X* and *Y* with respect to the state *ρ* and (Δ*X*)^2^ = Cov_*ρ*_(*X*, *X*) denotes the variance. The first term of 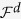 represents the contribution from the system and the second term represents that from the dissipative bath. 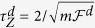 is called as the dissipative quantum Zeno time (d-ZT) which coincides with the largest interval such that the two states remain indistinguishable^17^. We can conclude that the larger d-FI is, the shorter d-ZT will be and the harder quantum Zeno dynamics is to be realized.

Then, we consider an exactly solvable model, the damped Jaynes-Cummings model (JCM)^22^, to study the Zeno dynamics in non-Markovian environments. A Hamiltonian of the total system is given by *H*_tot_ = *H*_*S*_ + *H*_*B*_ + *H*_*I*_ where the system’s Hamiltonian is *H*_*S*_ = *ω*_0_*σ*_+_*σ*_−_, the Hamiltonian of vacuum reservoir is 
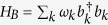
, and *H*_*I*_ = *σ*_+_*B* + *σ*_−_*B*^†^ denotes the interaction Hamiltonian given that *B* = ∑_*k*_*g*_*k*_*b*_*k*_ with *b*_*k*_ (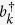) the boson annihilation (creation) operator for the *k*th mode. Here, *ω*_0_ denotes the transition frequency of the atom with ground state |0〉 and excited state states |1〉; *σ*_*x*,*y*,*z*_ are Pauli operators and *σ*_±_ are the raising and lowering operators. The initial state is given as 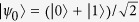. Given the Lorentzian spectral density 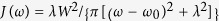 with *W* the transition strength and *λ* the spectral width of the coupling, we can obtain the master equation 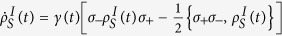 where the time-dependent decay rate *γ*(*t*) is written in two conditional forms:


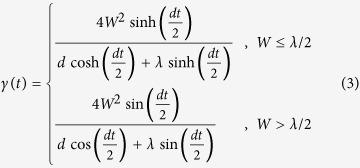


with 
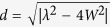
. In the weak coupling regime *W* < *λ*/2, *γ*(*t*) is always positive which corresponds to the Markovian process, while in the strong coupling regime *W* ≥ *λ*/2, the function *γ*(*t*) becomes negative within certain intervals of time, which displays the non-Markovianity^24^. For both Markovian and non-Markovian regimes, we obtain the same results as 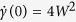. The d-FI is calculated as 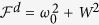 which leads to 
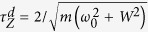
. If there is no noise *W* = 0, the result will reduce to the unitary evolution case as discussed in Ref. 17. When the transition strength becomes larger, d-FI grows and d-ZT decreases, which makes the Zeno dynamics more difficult. Moreover, given a definite value of transition strength *W*, d-FI is independent of *λ* and the result stays unchanged for both Markovian and non-Markovian noise. The interpretation of this extraordinary result may be that in this example the initial dynamics characteristics of the open system do not depend on Markovianity or non-Markovianity.

**Figure 1:**
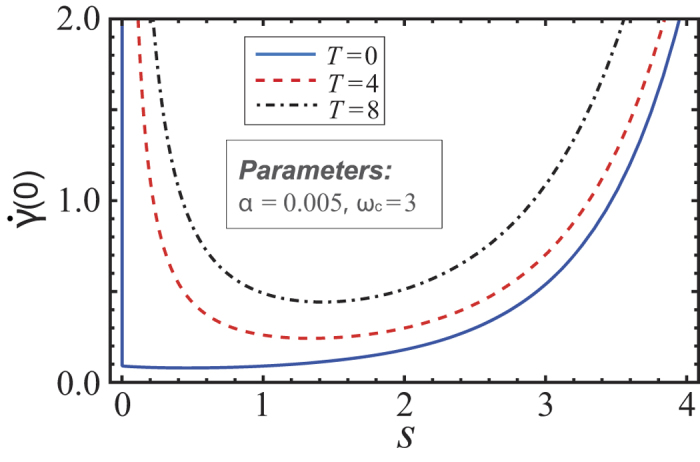
The first derivative of decay rate for *t* = 0 agianst *s*. Parameters are set as *α* = 0.01 and *ω*_*c*_ = 3. Three cases with three temperatures *T* = 0, 4 and 8 are plotted by blue solid line, red dashed line and black dot-dashed line, respectively.

Next, for the same initial state, we consider another exactly solvable model, the independent boson model, with *H*_*S*_ = *ω*_0_*σ*_*z*_/2, 
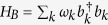
 and 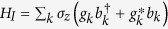. We consider the general Ohmic-like spectral density with exponential cutoff 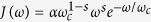: for *s* = 1 it is Ohmic; for *s* > 1 it is super-Ohmic; for *s* < 1 it is sub-Ohmic^22^. The bath is assumed to be initially in a thermal state: *ρ*_*B*_ = exp(−*H*_*B*_/*T*)/Tr[exp(−*H*_*B*_/*T*)] given *T* the temperature. Then we can obtain the master equation as 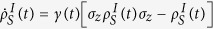 where 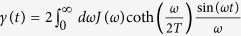. For zero temperature, the time-dependent decay rate can be carefully calculated as 
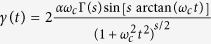
 where Γ(*s*) is the Euler Gamma function. If we consider non-zero temperature cases, the first derivative of decay rate for *t* = 0 can be exactly obtained as


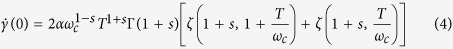


where 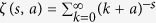 is the Hurwitz zeta function (a generalized Riemann zeta function). The d-FI and d-ZT may be calculated as 
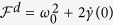
 and 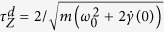, where first derivative of decay rate for *t* = 0 is shown in Fig. 1 given the parameters *α* = 0.005 and *ω*_*c*_ = 3. It is shown that, as the temperature of bath becomes higher, 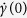 increases, which makes the quantum Zeno dynamics more difficult. Moreover, we find that for a definite temperature *T*, 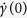 declines first and increases then as *s* increases. Thus, to realize the Zeno dynamics for this model depends on the temperature and the spectral density function of the bath.

### Quantum Zeno dynamics via operator-sum representation

A quantum process described in terms of an operator-sum representation is more general than the one written down as a master equation^21^. For most circumstances, the noisy quantum channel can be written as a quantum dynamical map 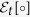 in terms of Kraus operators {Π_*l*_(*t*)} with time *t* the parameter^21^. The state evolves as 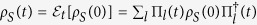, and the dynamical map is assumed to reduce to the identity map as *t* = 0. This nonunitary time evolution can also be transformed into a unitary time evolution operator on an enlarged space *S* + *E* for the system *S* interacting with an environment *E*. It can be expressed as 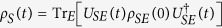, where the initial state of *S* + *E* is assumed to be initially decoupled *ρ*_*SE*_(0) ≡ *ρ*_*S*_(0)⊗|0〉_*E*_〈0| and the unitary time evolution operator is assumed to have property 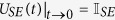 with 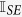 the identity of enlarged space *S* + *E*. Therefore, we are able to use the results of QZE of unitary time evolution discussed in Ref. 18 to investigate the quantum Zeno dynamics in open system.

After a sequence of *m* observations using measurement operator 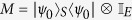 with 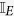 the identity of environment *E*, the state of the system *S* stays unchange with a survival probability 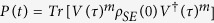, where *V*(*τ*) = *MU*_*SE*_(*τ*)*M* and still the interval is *τ* = *t*/*m*. For infinitesimal time intervals *τ* ≪ *t* with a large enough number of trials *m* → ∞, the survival probability can be expanded in terms of intervals *τ* (See Methods for details.)


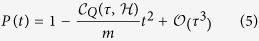


in which the Hermitian operator 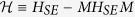 and the Hermitian generator of displacement in parameter *t* is^25^ *H*_*SE*_ ≡ −*i*[*dU*_*SE*_(*t*)/*dt*]_*t* → 0_. Here, 
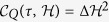
 can be regarded as the QFI for the Hermitian generator 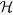 of the enlarged system *S* + *E*. The information about the interval *τ* when system *S* and environment *E* are monitored together is larger or equal to that obtained when merely the system *S* is monitored. Therefore, the QFI of the enlarged system *S* + *E* gives an achievable upper bound of the QFI of system *S*^19^,^20^: 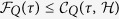. The ZT of the enlarged system *S* + *E* has a time scale that is upper bounded by the smallest path interval of QFI of system alone such that two states are statistically distinguishable^16^,^18^


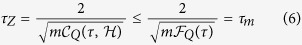


where *τ*_*m*_ is the largest interval for QZE of the noisy quantum channel. The achievable maximum of *τ*_*Z*_ is obtained when 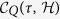 reaches its minimum, which is tantamount to calculating the QFI of a noisy quantum channel 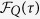 corresponding to the entire unitary time evolution 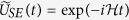
20.

The QFI of a noisy quantum channel can be achieved over all the possible and effective operator 
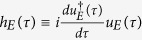
 with unitary operator *u*_*E*_(*τ*) acting solely on the space of environment *E*^20^,^21^ (see Methods for details). Therefore, we can obtain^20^


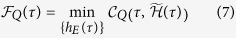


where we define the Hermitian operator as 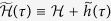 and 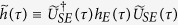. The unitary time evolution *u*_*E*_(*t*)*U*_*SE*_(*t*) of environment *E* together with system *S* does not lead to more information about parameter *τ* than that obtained by system *S* itself. We are also able to find a set of equations for the optimum effective Hermitian operator 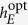 that minimizes 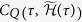.

There are, in fact, infinite different unitary evolutions of the enlarged system *S* + *E* corresponding to the same operator-sum representation of system *S*, since it has the unitary freedom *u*_*E*_(*t*)^21^,^26^. Each one gives a different value of QFI 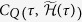. Even so, the maximum ZT *τ*_*m*_ leads to an interesting and important physical insight: there is always an environment *E* making the quantum Zeno dynamics most accessible. This result is promising to protect quantum information from decoherence, especially for entangled states.

### *N*-qubit quantum Zeno dynamics in quantum open systems

Different states, entangled or separable, with different values of QFI lead to different ZT scales^18^. QFI of a separable state of an *N*-qubit system governed by a local Hamiltonian 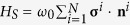, is bounded by 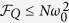, where 
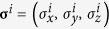
 is the vector of three Pauli matrices acting on the *i*-th qubit and ***n***^*i*^ is a unit vector. Therefore, 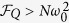 is a sufficient condition for the presence of entanglement^17^. As a consequence of larger QFI, the quantum Zeno dynamics of entangled states may require a much higher rate of projective measurements than that of separable ones as the number of qubits grows too large^18^. Next, we will exemplify that the quantum Zeno dynamics of maximal entangled states may only require a similar number of measurements as that of separable states if the system interacts with a proper environment.

We consider an *N*-qubit system of which each qubit merely interacts with a corresponding environmental qubit. It can be described as a unitary operator onto an enlarged system *S* + *E* by tracing out all the environmental qubits:


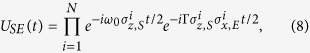


where 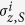 is the Pauli matrix acting on the *i*th system qubit and 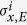 is on its environment qubit. The initial state of the environment qubits is set as 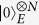. Given the system’s initial state a maximal entangled state 
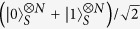
, we can obtain the QFI as (See Methods.):


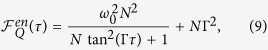


which has a limit 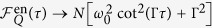 as *N* → ∞. It leads to the upper bound of ZT 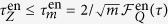. The QFI of a separable initial state of the system with form 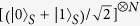 for the same quantum dynamical map is


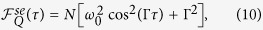


and 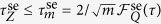. We can conclude that the ratio 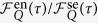 is independent of *N* for an infinitely great *N*, no matter how small the interaction between system and environment is. This result conforms to the conclusions of entanglement-enhanced parameter estimation in open systems: the use of maximal entangled states fails to provide higher resolution as compared to using separable states where decoherence exists^27^,^28^,^29^,^30^,^31^.

**Figure 2:**
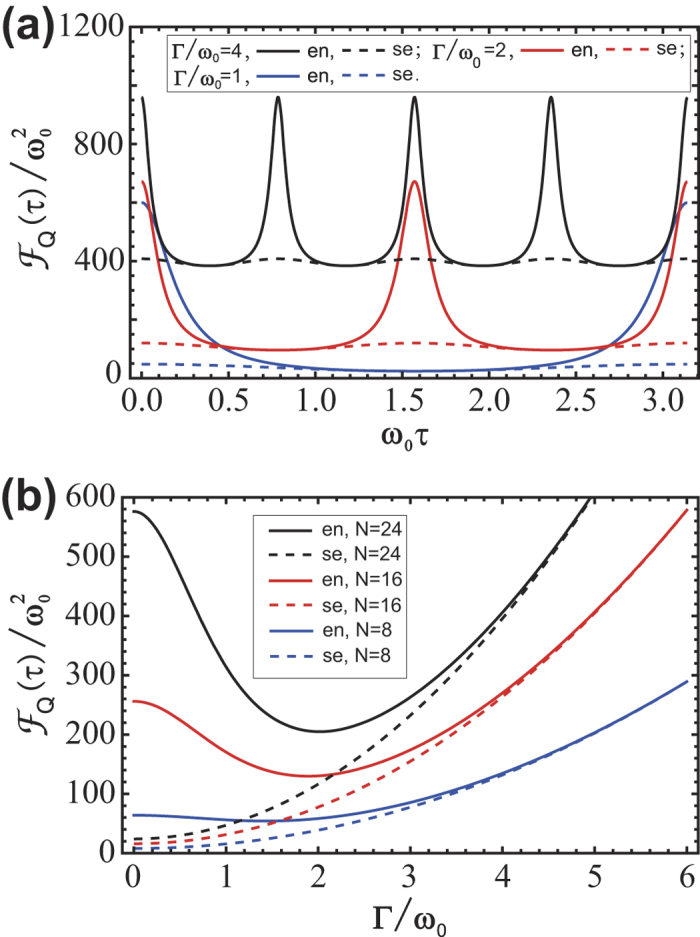
QFI of separable state and entangled state. Solid lines are for QFI of entangled state (en) and dashed lines are for separable state (se). (**a**) 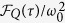 against *ω*_0_*τ* with different interaction strength: Γ/*ω*_0_ = 1 (blue lines), 2 (red lines) and 4 (black lines). The qubit number is set as *N* = 24. (**b**) 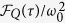 against Γ/*ω*_0_ with different qubit numbers: *N* = 8 (blue lines), 16 (red lines) and 24 (black lines). The time interval is set as *ω*_0_*τ* = 0.2.

Specifically, we can obviously see from Fig. 2(a) that 
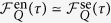
 for some time interval. In Fig. 2(b) we find that when the strength of environment Γ is weak, 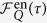 is larger than 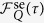, i.e., quantum Zeno dynamics of entangled states may be harder to realize. As the increase of Γ, quantum Zeno dynamics of both cases are equally accessible. However, given “strong environment” Γ/*ω*_0_ ≪ 1, both 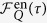 and 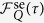 tend to be infinity and the ZT is confined to be so small that it makes the quantum Zeno dynamics nearly accessible as predicted in Ref. 18. It is thus significant that the appropriate environmental interaction can be favourable for realizing QZE of entangled states compared with the case of unitary time evolution in closed system. This effect may be explained by the fact that some decoherence acts like an effective further continuous measurement on the system, therefore making the QZE more accessible. Besides, we can also figure out the optimal model of environment given the definite form of states and the definite noisy channel, which is shown in Methods. Our theory is also able to settle the case for states which are not maximally entangled but may bring new interesting results of QZE in open system.

## Discussion

We have investigated the accessibility of quantum Zeno dynamics in quantum open systems. The quantum Zeno dynamics in non-Markovian noise has been studied with d-FI and d-ZT when the exact master equations are used to describe the quantum open system. The more general description using operator-sum representation of the open system has also been considered and investigated. Due to the external unitary freedom of this description, an achievable upper bound of ZT is deduced via the variational methods. Although entanglement will enhance the speed of evolution and hinder QZE for unitary process^32^, we have been examplified that the quantum Zeno dynamics of maximal entangled states can be realized much easier when they interacts with the proper environment than without noise. That is, the noise reducing the accuracy in the quantum parameter estimation can conversely be favorable for the accessibility of quantum Zeno dynamics of entangled states. Our work will help to stablize the system of entangled states against time evolution and noise in many quantum systems^7^,^9^,^12^,^31^.

## Methods

### QFI and Zeno dynamics

Given a unitary dynamics *e*^−*iHt*^|*ψ*_0_〉 and *m* trials of projections *M* = |*ψ*_0_〉〈*ψ*_0_| = *ρ*_0_, the survival probability of the Zeno dynamics is 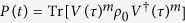 where *τ* = *t*/*m* and *V*(*τ*) ≡ *Me*^−*iHτ*^*M*. The survival probability for small time intervals *τ* can be expanded as 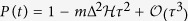 where 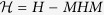. The leading role in the theory of this work is played by the FI^33^: 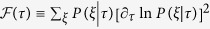 where *P*(*ξ*|*τ*) = Tr[*ρ*(*τ*)*E*(*ξ*)] given {*E*(*ξ*)} a set of POVMs. QFI is obtained by exploiting the maximum of FI among all the possible POVMs, and for unitary evolution 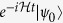, it can be expressed in a simple analytical expression^34^: 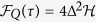 when we take *t* as the unknown parameter. Therefore, the Zeno dynamics for unitary evolution with generator *H* can be approximately described by the QFI of the unitary evolution with Hermitian generator 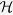.

Generally, if the survival probability for intevals, *τ* ≪ 1, can be expanded as 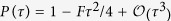, we can calculate the FI as 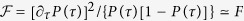. For the master equation approach, the survival probability 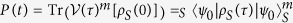 and we can expand the density operator 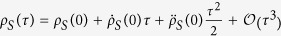 for small time intervals. We can obtain the first order derivative as 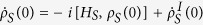 and for most physical cases without the Markovian approximation, *γ*_*k*_(0) = *S*_*k*_(0) = 0 and 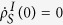 hold for all spectral densities^22^,^27^,^35^ with which we obtain 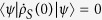. For the second order derivative, we have 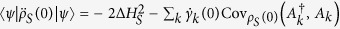, with which Eq. (1) can be proved.

### Noisy QFI of maximal entangled state of *N* qubits

In the noisy model expressed in Eq. (8), the Hermitian operator may be calculated as 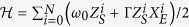 such that 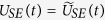. In accordance with the symmetry of maximal entangled states, the general form of the Hermitian operator acting solely on environment *E* may be expressed as^36^


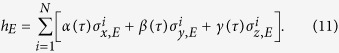


where *α*(*τ*), *β*(*τ*) and *γ*(*τ*) are variables in terms of parameter *τ*. We can calculate the exact form of 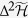 as


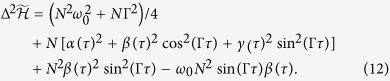


Then, we minimize 
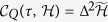
 over *α*(*τ*), *β*(*τ*) and *γ*(*τ*) for any value of *τ* with conditions 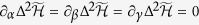. The optimal parametric equations may be obtained as *α*^opt^(*τ*) = *γ*^opt^(*τ*) = 0 and *β*^opt^(*τ*) = (*ω*_0_*N*sin(Γ*τ*))/(2[*N*sin^2^(Γ*τ*) + cos^2^(Γ*τ*)]). Thus, the QFI of the noisy system 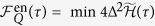 is obtained as shown in Eq. (9). For the separable state, we let *N* = 1 and obtain the QFI using the additivity of FI.

With the optimal parametric equations, we are still able to obtain the exact form of the optimal environment that maximizes the ZT. With the optimal Hermitian operator 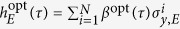, the optimal unitary time evolution is written as 
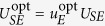
 with 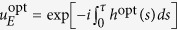.

## Additional Information

**How to cite this article**: Zhang, Y.-R. and Fan, H. Zeno dynamics in quantum open systems. *Sci. Rep.* **5**, 11509; doi: 10.1038/srep11509 (2015).
